# Supplementary material for: ProteinShader: illustrative rendering of macromolecules
Source: BMC Struct Biol. 2009 Mar 30;9:19. doi: 10.1186/1472-6807-9-19 (PMC2672931; doi:10.1186/1472-6807-9-19)
Supplement: Additional file 1 — ProteinShader program without source code. This compressed file contains the complete ProteinShader program including associated libraries, but no source code. A README.txt file gives an overview of the ProteinShader distribution, and the index.html file in the help subdirectory has directions on getting started with the program as well as a set of tutorials. [file 1472-6807-9-19-S1.zip › ProteinShader-beta-0_9_4-binary/help/api/org/proteinshader/structure/enums/class-use/RegionEnum.html]

Uses of Class org.proteinshader.structure.enums.RegionEnum (ProteinShader API)


|  |  |  |
| --- | --- | --- |
| **Overview** \| **Package** \| **Class** \| **Use** \| **Tree** \| **Deprecated** \| **Index** \| **Help** | |  |
| PREV   NEXT | **FRAMES**    **NO FRAMES**     **All Classes** |  |


---


## **Uses of Class org.proteinshader.structure.enums.RegionEnum**

| Packages that use RegionEnum | |
| --- | --- |
| **org.proteinshader.graphics** | Holds the drawing classes: Ribbon, Tube, FrenetFrames, Sphere, and Cylinder. |
| **org.proteinshader.graphics.typography** | Holds the classes needed for applying text labels to the curved surfaces of tubes and ribbons. |
| **org.proteinshader.structure** | Holds the classes that store information from a Protein Data Bank file: Structure, Model, Chain, AminoAcid, Heterogen, Water, Atom, Bond, Helix, BetaStrand, Loop, *etc*. |
| **org.proteinshader.structure.enums** | Holds the enums needed by org.proteinshader.structure and its subpackages. |
| **org.proteinshader.structure.io** | Holds the classes needed for reading Protein Data Bank structure files. |

Uses of RegionEnum in org.proteinshader.graphics

Constructors in org.proteinshader.graphics with parameters of type RegionEnum

`Ribbon(RegionEnum regionType)`
  
          Constructs a Ribbon using default values based on the Region type
(Helix, BetaStrand, or Loop).

`Tube(RegionEnum regionType)`
  
          Constructs a Tube object that can be used for rendering tubes with
a circular-shaped waist polygon.

Uses of RegionEnum in org.proteinshader.graphics.typography

| Methods in org.proteinshader.graphics.typography with parameters of type RegionEnum | |
| --- | --- |
| `void` | `TextLabelManager.enableTexture(GL gl, String segmentID, int shaders, RegionEnum regionType)`             Enables OpenGL texture mapping and binds a texture with text cooresponding to the segmentID given as an argument. |

Uses of RegionEnum in org.proteinshader.structure

| Methods in org.proteinshader.structure that return RegionEnum | |
| --- | --- |
| `RegionEnum` | `AminoAcid.getRegionType()`             Returns the region type (LOOP, HELIX, or BETA\_STRAND) if the AminoAcid has been assigned to a Region. |
| `RegionEnum` | `Segment.getRegionType()`             Returns the type of Region that this Segment belongs to. |

| Methods in org.proteinshader.structure with parameters of type RegionEnum | |
| --- | --- |
| `Segment` | `Model.getSegment(String segmentID, String regionID, RegionEnum regionType)`             Returns the requested Segment if it exists. |
| `protected  void` | `Region.markAminoAcids(String regionID, RegionEnum regionType)`             Used to mark each AminoAcid with the ID and type of Region it belongs to. |
| `void` | `AminoAcid.setRegionIDAndType(String regionID, RegionEnum regionType)`             Sets the regionID and the Region type. |

Uses of RegionEnum in org.proteinshader.structure.enums

| Methods in org.proteinshader.structure.enums that return RegionEnum | |
| --- | --- |
| `static RegionEnum` | `RegionEnum.valueOf(String name)`             Returns the enum constant of this type with the specified name. |
| `static RegionEnum[]` | `RegionEnum.values()`             Returns an array containing the constants of this enum type, in the order they're declared. |

Uses of RegionEnum in org.proteinshader.structure.io

Constructors in org.proteinshader.structure.io with parameters of type RegionEnum

`TubeVertices(RegionEnum regionType)`
  
          Constructs a TubeVertices object that can be used for rendering
tubes with a circular-shaped waist polygon.

---


|  |  |  |
| --- | --- | --- |
| **Overview** \| **Package** \| **Class** \| **Use** \| **Tree** \| **Deprecated** \| **Index** \| **Help** | |  |
| PREV   NEXT | **FRAMES**    **NO FRAMES**     **All Classes** |  |


---

# *Copyright © 2007-2008*
